# Symbiotic strategies: deciphering the role of gut microbiota in the nutrition and metabolism of fish and shellfish

**DOI:** 10.3389/fcimb.2025.1639426

**Published:** 2025-10-14

**Authors:** Nandini Rai, Ankit Kachore, J. M. Julka, Akshaya Panigrahi, Sofia Priyadarsani Das, Fan-Hua Nan

**Affiliations:** ^1^ School of Biological and Environmental Sciences, Shoolini University of Biotechnology and Management Sciences, Solan, Himachal Pradesh, India; ^2^ School of Advanced Chemical Sciences, Shoolini University of Biotechnology and Management Sciences, Solan, Himachal Pradesh, India; ^3^ Crustacean Culture Division, Indian Council of Agricultural Research (ICAR)-Central Institute of Brackish-water Aquaculture, Chennai, Tamil Nadu, India; ^4^ College of Life Sciences, Department of Aquaculture, National Taiwan Ocean University, Keelung, Taiwan

**Keywords:** aquaculture sustainability, environmental factors, gut microbiota, innate immunity, metabolism, microbial diversity, nutrition

## Abstract

The gastrointestinal microbiota is crucial for the health and physiology of aquatic organisms, influencing their nutrition, metabolism, and immune responses. This review compares the diversity and function of gut microbial communities in finfish and shellfish, highlighting differences between freshwater and marine species as well as variations within shellfish taxa. We examine how these microbes aid in digesting complex dietary substrates, assimilating nutrients, and synthesizing essential metabolites, all of which are vital for host health. The structure of these microbial communities is shaped by a complex interplay of environmental factors, such as water temperature, salinity, and pH, and host-specific factors, including genetics and diet. A comprehensive understanding of these interactions is key to improving gut health and nutrient use in aquaculture. This review also identifies future research directions, focusing on the use of probiotics, prebiotics, and dietary interventions. These strategies, combined with multi-omics approaches, have great potential to enhance the sustainability of aquaculture by improving growth performance, feed conversion efficiency, and disease resistance in farmed aquatic species.

## 1 Introduction

Diverse polymicrobial communities—including bacteria, archaea, viruses, yeasts, and protists are ubiquitously associated with aquatic organisms, colonizing niches such as the gastrointestinal tract, skin, gills, and muscle tissues (Li et al., 2022). Their composition and abundance are shaped by environmental factors and host-specific traits like genetics, developmental stage, sex, and diet. Despite this variability, a core gut microbiota often persists across conspecifics, reflecting adaptation to host-specific selective pressures (Diwan et al., 2021). These microbial communities are essential for maintaining physiological homeostasis, supporting nutrient assimilation, immune function, and defense against pathogens. The development of the digestive system and immune modulation are closely tied to the presence of these microbes (Li et al., 2022; Merrifield and Rodiles, 2015). Recent research, including single-cell analyses, highlights the molecular complexity of host microbe interactions and their critical roles in health and disease outcomes (Sharma and Thaiss, 2020).

The study understanding of the complicated internal workings of organisms has been considerably improved by breakthroughs in single-cell analytical tools, particularly those that examine genes, RNA messages, and the spatial organization of cells (Sharma and Thaiss, 2020). While the profound influence of host-microbiota interactions on host development, immunity, metabolism, and associated signalling pathways is now well-established, specific knowledge regarding these interactions in fish and shellfish remains comparatively limited (Sharma and Thaiss, 2020). However, recent research endeavours have begun to elucidate the nature of host-microbiota interactions in the context of various physiological functions within these aquatic organisms. One study investigated these interactions in hybrid fish derived from parental lineages exhibiting contrasting herbivorous and carnivorous dietary adaptations (Pérez et al., 2010). Their findings indicated comparable growth trajectories during early ontogeny and minimal divergence in microbial composition at this initial developmental stage. However, significant alterations in microbiota structure were observed during subsequent developmental phases, coinciding with shifts in dietary regimes. Subsequent analyses revealed a predominance of microbial species associated with metabolic processes and growth in both dietary groups. Furthermore, differentially expressed homologous genes within the intestine, linked to cellular proliferation, immune responses, and metabolic pathways, exhibited correlation with the dominant gut microbiota taxa, suggesting host genes and gut microbes likely work together to help these hybrid fish adapt to their diet (Pérez et al., 2010). Investigations have also focused on the microbial populations within fish digestive systems and their relationships with the mucosal layers (Merrifield and Rodiles, 2015). The research team led by Nerea Arias Jayo also investigated how zebrafish’s gut bacteria changed when the fish ate a diet high in saturated fats with added fish oil. Their work emphasized how important what an animal eats is for the relationship between the animal and the microbes living in its gut (Arias-Jayo et al., 2019). Moreover, research analyzing the gut bacteria of five marine fish species reared together suggested that this intestinal microbial community functions like a second set of genes, influencing various vital physiological processes (Nikouli et al., 2021). The study also reported that a fish gut bacterial community develops in response to both internal factors and external environmental conditions, including diet (Nikouli et al., 2021).

Many studies have elucidated the functional roles of gut microbiota and their interactions with fish and shellfish hosts, including their involvement in digestive enzyme production (Ray et al., 2012) and processes like vitamin synthesis, short-chain fatty acid production, biofilm formation, and iron metabolism in freshwater and marine fish (Romero et al., 2014; Tsuchiya et al., 2008; Xing et al., 2013). The study thoroughly investigated the connection between gut microbiota and growth performance in hybrid fish (Li et al., 2022). While fewer studies have assessed phenotypic variations in fish and shellfish in correlation with gut microbiota, “the species composition of the gut microbiota has been analyzed to determine the involvement of microbial genomes in the selection of core microbiota members (Mushegian et al., 2019). Studies have also examined how the relationship between fish and their gut microbes helps manage stress in different types of fish (Cui et al., 2022; Mohanta et al., 2020). In the context of shellfish, particularly penaeid shrimp, the available information regarding host-microbiota interactions is comparatively limited. Thorough investigation has shown that disease-causing microbes exist in different tissues of penaeid shrimp, negatively impacting their health and the output of aquaculture (Chaiyapechara et al., 2021). However, the burgeoning appreciation for the microbiota’s role in bolstering physiological functions has highlighted its contributions to enhanced immunity and healthy growth. Therefore, to effectively use the shrimp gut microbiota for improving overall health and quality, it’s essential to grasp how external and internal elements affect its composition. Moreover, the study explored how the community of microbes present in the gut of black tiger shrimp (*Penaeus monodon*) changes, and how their gene activity varies when the shrimp are exposed to different salinities (Chaiyapechara et al., 2021). Their findings indicated that shrimp acclimatized to higher salinities exhibited a gut microbiota dominated by the phylum Proteobacteria, followed by Bacteroidetes and Planctomycetes. The most prevalent genus was *Vibrio*, belonging to the Harveyi species. Furthermore, they reported differential expression of genes associated with stress and immunity at higher salinities, as the abundance of pathogenic *Vibrio* increases, expression of genes related to the host’s innate immune response also increases. Other studies have also noted comparable effects of salinity on the bacteria in shrimp intestines (Angthong et al., 2020; Fan et al., 2019; Huang et al., 2016; Rungrassamee et al., 2013). This study looked more closely at the part gut bacteria play in keeping shrimp healthy and controlling diseases. It proposed that changing the gut’s microbial balance by giving shrimp beneficial microbes can have a positive impact on their growth and survival rates (Holt et al., 2020). Recognizing the crucial roles of gut microbiota in shrimp health and immunity (Ma et al., 2018; Tarnecki et al., 2017), an alternative approach for preventing diseases and enhancing shrimp well-being involves strategically modifying the gut microbial community to encourage the growth of beneficial bacteria. Given that factors like culture conditions, developmental phases, and health status can alter shrimp gut microbial composition (Angthong et al., 2020; Fan et al., 2019; Huang et al., 2016; Zheng et al., 2017), a well-defined research strategy is essential to understand these intricate relationships. Researcher investigated the relationship between growth performance in *P. monodon* and gut microbiota composition, transcriptome, and metabolites, reporting a relative surfeit of bacteria such as *Brevibacillus* and *Spongiimonas* in the majority of shrimp guts (Uengwetwanit et al., 2020). Researchers have also identified distinct gene activity patterns in the intestines and specific immune-related genes in shrimp showing different growth rates. Recent progress in studying the microbiota and gene expression in shrimp after probiotic feeding has revealed a strong connection between the types of gut bacteria present and the host’s gene activity related to immunity, digestion, and programmed cell death (Duan et al., 2018, 2019b, 2019a). Researchers investigating the gut bacteria of different penaeid shrimp species have emphasized the crucial part played by the relationships between the host and its microbes in elucidating the basic mechanisms that underpin a wide range of bodily functions (Imaizumi et al., 2021; Dai et al., 2018; Zoqratt et al., 2018).

**Figure 1:**
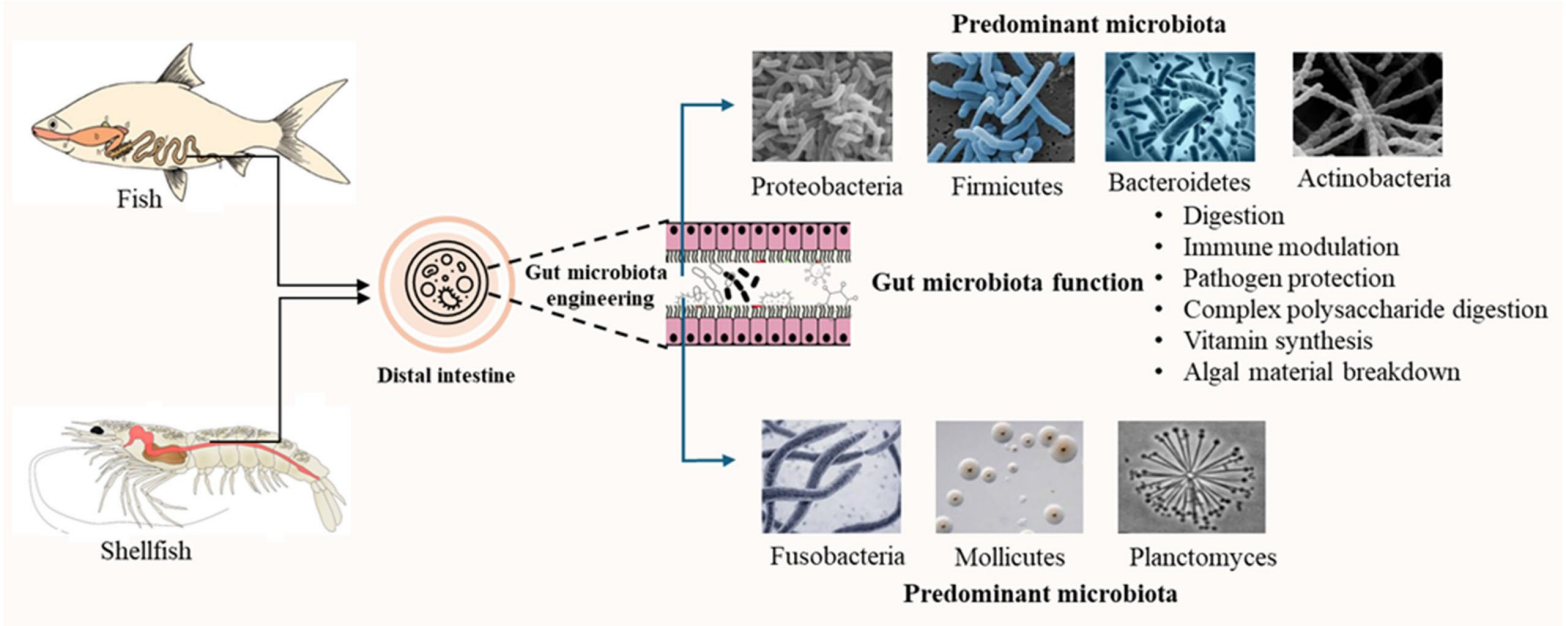
An illustrated overview of the gut microbiota roles and functions in fish and shellfish.

This review synthesizes current knowledge on the complex interactions between host organisms and their gut microbiota in commercially important fish and shellfish species. It critically evaluates the role of gut microbial communities in key physiological processes, with a focus on digestive efficiency, nutrient assimilation, and metabolic regulation. The review first explores the diversity and composition of gut microbiota across freshwater and marine fish, as well as various shellfish species. It then examines the functional contributions of these microbes to host metabolism and analyses the environmental, genetic, and dietary factors that shape microbial diversity and function. Adopting a multidisciplinary perspective, this review highlights the potential of microbiota-driven strategies to enhance aquaculture productivity, support host health, and promote sustainable aquatic food systems (**Figure 1**).

## 2 Diversity and composition of gut microbiota in fish and shellfish

The complex and dynamic microbiota inhabiting the fish gastrointestinal tract exerts a significant influence (Zhang et al., 2021). A stable state characterized by a harmonious interaction between the host and gut microorganisms is vital for healthy intestinal function (Banerjee and Ray, 2017). In fact, these resident bacterial populations significantly influence numerous physiological processes, such as maintaining equilibrium, growth, nutrient processing, reproduction, and immune responses (Butt and Volkoff, 2019). Acknowledging the significance of gut flora for the health of fish, including their immune system, physical functions, and overall well-being (Yang et al., 2021), and given that an unbalanced gut microbiota is associated with internal instability and disease development (Wang A, et al., 2018), current scientific research is focused on the potential of using prebiotics, probiotics, and symbiotic to alter the gut microbiota as a way to improve fish health (Nguyen et al., 2018).

Studies have shown that the Bacteroidetes phylum is important for boosting the natural defences system of fish (Gómez and Balcázar, 2008) and for changing the host immune responses, which helps protect them from diseases (Talwar et al., 2018). Furthermore, the Bacteroidetes phylum exhibits a notable metabolic capability to catabolize complex polysaccharides into simpler monosaccharide units (Xu et al., 2003). The development of fast and affordable high-throughput sequencing has dramatically changed how we measure and describe the makeup of microbial communities. This has greatly improved our understanding of the microbiota and made it possible to thoroughly study the bacteria living in fish guts (Chen et al., 2022). Recent advancements in ichthyic microbiota research have garnered considerable attention, recognizing the microbiota a complex consortium encompassing bacteria, fungi, and viruses (Morshed and Lee, 2023) as a critical determinant of host health and homeostasis. Correspondingly, a growing understanding of the microbiota vital function within the host physiology, representing a significant area of application within holobiont research, which focuses on the prevention and therapeutic intervention of diseases through targeted modulation aimed at restoring dysbiotic microbial communities (Diwan et al., 2023). The phylum Actinobacteria, the most extensive prokaryotic group, predominantly comprises Gram-positive bacterial species exhibiting a diverse array of morphological and developmental characteristics (Bhatti et al., 2017). Notably, comparative analyses have identified Bacteroidetes and Firmicutes as potential biomarkers for assessing lipid metabolism in *Cyprinus carpio* (Meng et al., 2018). A specific study (Talwar et al., 2018) indicated a positive correlation between the phylum Firmicutes and accelerated growth rates in fish compared to the phylum Bacteroidetes. Geerlings et al., 2021 research suggests that bacteria belonging to the phylum verrucomicrobia play a role in breaking down mucus in fish digestion. Recent scientific discoveries highlight the significant influence of gut microbes on the proper development of reproductive systems and subsequent successful reproduction in fish. When zebrafish (*Danio rerio*) were given *Lactobacillus rhamnosus*, a type of bacteria in the Firmicutes phylum, from hatching until they reached sexual maturity, their gut bacteria changed, and they developed faster. This was likely due to enhanced growth and the processes that determine their sex (Avella et al., 2012; Carnevali et al., 2013). Conversely, Bozzi et al., 2021 identified *Tenacibaculum dicentrarchi* as a pathogenic bacterial strain and observed a distinct microbial profile in the distal gastrointestinal tract (GIT) of diseased fish compared to their healthy counterparts. Early exposure of newly hatched fish larvae to commensal microbiota present in the aquatic environment likely confers a protective advantage against opportunistic pathogenic bacteria such as *Aeromonas hydrophila* (Brugman et al., 2018).

### 2.1 Freshwater fish gut microbiota

Freshwater investigations have revealed that the core gut bacteria in *Oncorhynchus mykiss* are resilient to environmental shifts such as different diets and varying stocking densities (Wong et al., 2013), suggesting a stable microbial community. Nevertheless, alterations in diet can still impact the host’s health status (Wong et al., 2013). In herbivorous and omnivorous fish, the enhanced breakdown of cellulose has been linked to the presence of specific bacterial genera, namely *Bacillus circulans* and *B. megaterium* (Saha et al., 2006). Investigations into the gut microbiota ontogeny of *Carassius auratus gibelio* have identified Proteobacteria as the initially dominant phylum colonizing the gastrointestinal tract (Li et al., 2017); this early dominance may be due to their widespread distribution in aquatic environments, facilitating early host–microbe interactions. Beyond Proteobacteria, other commonly found bacterial phyla in freshwater ecosystems include Actinobacteria, Bacteroidetes, Cyanobacteria, and Firmicutes (Cottrell et al., 2005; Jordaan and Bezuidenhout, 2013; Savio et al., 2015; Brar et al., 2023). According to Wu et al. (2012), the most prevalent bacterial group in the gut of freshwater fish is typically Proteobacteria, followed by Firmicutes, Actinobacteria, and Bacteroidetes. Actinobacteria are known for producing a diverse array of secondary metabolites, including hydrolytic enzymes that break down complex molecules, and for contributing to the fermentation of oligosaccharides (Ventura et al., 2007). Members of the phylum Fusobacteria are also frequently detected in freshwater fish (Kim et al., 2021). Common genera and species found include *Enterobacter, Aeromonas, Acinetobacter, Escherichia, Klebsiella, Proteus, Serratia, Alcaligenes, Listeria, Bacillus, Bacteroides, Staphylococcus*, and *Pseudomonas* (Austin, 2002; Brown et al., 2019; Hernández et al., 2021; Singh et al., 2021) (**Table 1**).

**Table 1:** Gut microbiota composition and function in freshwater and Marine water fish species.

Species	Feeding Behaviour	Habitat	Predominant microbiota	Key Genera	Microbial Roles	Cited Studies
*Oncorhynchus mykiss* (Rainbow Trout)	Carnivorous (aquatic insects, crustaceans, small fish)	Cold water (10-15°C)	Proteobacteria, Firmicutes, Bacteroidetes, Actinobacteria	*Aeromonas, Pseudomonas, Acinetobacter, Clostridium, Lactococcus*	Digestion, immune modulation, pathogen protection, complex polysaccharide digestion, vitamin synthesis	Wong et al., 2013; Llewellyn et al., 2014; Betiku et al., 2023; Mondal et al., 2008; Li et al., 2016; Podell et al., 2023; Bao et al., 2023
*Ctenophardonie-a* (Grass Carp)	Herbivorous (aquatic plants)	Warm water (20-30°C)	Firmicutes, Proteobacteria, Actinobacteria, Bacteroidetes	*Aeromonas, Bacillus, Clostridium, Bacteroides, Lactobacillus, Flavobacterium, Veillonella*	Fiber breakdown, nutrient production from plant material	Wu et al., 2012; Llewellyn et al., 2014; Ray et al., 2012
Carassius-auratus gibelio	Omnivorous	Freshwater	Proteobacteria, Firmicutes, Actinobacteria, Bacteroidetes	*-*	Ubiquitous interactions with host, nutrient cycling, organic matter breakdown	Li et al., 2017
Marine Fish (General)	Carnivorous and Herbivorous	Marine	Firmicutes, Proteobacteria, Actinobacteria	*Vibrio, Pseudomonas, Achromobacter, Corynebacterium, Flavobacterium, Micrococcus, Aeromonas, Alcaligenes, Alteromonas, Carnobacterium,Photobacterium*	Salt tolerance, nutrient absorption, digestion, pathogen protection	Austin, 2002; Izvekova et al., 2007; Huang et al., 2020; Ou et al., 2021
*Salmo salar* (Atlantic Salmon)	Carnivorous (marine animals)	Cold, well-oxygenated waters (anadromous)	Proteobacteria, Firmicutes, Actinobacteria	*Pseudomonas, Janthinobacterium, Burkholderia, Sphingomonas, Acinetobacter, Propionibacterium*	Nutrient absorption, digestion, immune modulation	Llewellyn et al., 2014; Gajardo et al., 2016; Morales et al., 2022; Moore et al., 2006; Wang C et al., 2018
*Acanthurus triostegus* (Surgeonfish)	Herbivorous (algae)	Warm tropical marine waters	Firmicutes, Proteobacteria, Actinobacteria	*Epulopiscium, Acinetobacter, Arcobacter, Arthrospira, Fusobacterium, Vibrio, Photobacterium*	Algal material breakdown, nutrient absorption	Miyake et al., 2016; Ngugi et al., 2017; Parata et al., 2020
*Pufferfish*	Omnivorous	Freshwater	Gammaproteobacteria, Fusobacteria, Actinobacteria, Anaerolineae, Betaproteobacteria, Deinococci, Clostridia, Deltaproteobacteria; Archaea: Methanomicrobia, Hadesarchaea, Thermoplasmata, Candidatus Altiarchaeales, Candidatus Bathyarchaeota, Thermoprotei	*Aeromonas, Plesiomonas, Cetobacterium*	Cofactor, vitamin, pigment, amino acid, carbohydrate and protein metabolism; similarities to carnivorous salmon (protein metabolism) and herbivorous grass carp (carbohydrate metabolism); trophic-level influence on gut microbiota	Deb et al., 2020
*Atlantic salmon (Salmo salar*	Carnivorous (marine animals	Cold Arctic waters; freshwater–seawater transition	Core taxa include Proteobacteria, Firmicutes, Actinobacteria (dynamic shifts across FW→SW stages)	*Pseudomonas, Janthinobacterium, Burkholderia, Sphingomonas, Acinetobacter, others varying by stage*	Seasonal and life-stage adaptation of gut microbiota; influenced by functional dietary ingredients (nucleotides, yeast cell walls, prebiotic, essential fatty acids); roles in nutrient absorption, digestion, immune modulation	Wang J et al., 2021
*Rohu (Labeo rohita*	Herbivorous (aquatic plants, detritus)	Freshwater aquaculture	Proteobacteria, Actinobacteria; core OTUs include *Streptomyces*	*Streptomyces and other core generalists;* sp*ecialists vary with culture condition*	Nutrient absorption, enzyme activity, probiotic potential; influenced by DO (negative correlation with diversity) and phosphate (positive correlation with Actinobacteria abundance); environmental modulation of gut community structure and function	Maji et al., 2022

A comparative analysis of *O. mykiss* (rainbow trout) and *Ctenopharyngodon idella* (grass carp) underscores the influence of trophic ecology and habitat on microbial composition. Rainbow trout, a carnivorous freshwater teleost that consumes aquatic insects, shellfish, and small fish (Huyben et al., 2018), prefers cooler waters (10–15 °C) and harbors gut microbiota dominated by Proteobacteria, Firmicutes, Bacteroidetes, and Actinobacteria, with *Aeromonas, Pseudomonas, Acinetobacter, Clostridium*, and *Lactococcus* prevalent at the genus level (Llewellyn et al., 2014; Betiku et al., 2023). These bacteria contribute to digestion, immune modulation (Mondal et al., 2008), pathogen defense, carbohydrate breakdown, and vitamin synthesis (Li et al., 2016; Podell et al., 2023). In contrast, grass carp—a herbivorous fish preferring warmer waters (20–30 °C; Ray et al., 2012)—hosts gut microbiota rich in *Aeromonas, Bacillus, Clostridium, Bacteroides*, and *Leuconostoc* (Wu et al., 2012; Llewellyn et al., 2014), reflecting adaptation to plant fiber degradation and plant-derived nutrient assimilation.

### 2.2 Marine water fish gut microbiota

The elevated salinity of marine environments imposes selective pressures on microbial communities, leading to greater heterogeneity in the gut microbiota of marine fish. Proteobacteria and Actinobacteria are the predominant phyla, although subgroups vary among species. Commonly occurring genera include *Vibrio, Flavobacterium, Micrococcus, Aeromonas, Alcaligenes, Alteromonas, Carnobacterium*, and *Photobacterium* (Izvekova et al., 2007; Huang et al., 2020; Ou et al., 2021). Comparisons between Atlantic salmon (*Salmo salar*) and surgeonfish highlight how habitat and diet shape microbial populations. Atlantic salmon, an anadromous carnivore thriving in cold, oxygen-rich waters, exhibits gut microbiota dominated by *Pseudomonas, Janthinobacterium, Burkholderia, Sphingomonas, Acinetobacter*, and *Propionibacterium* (Llewellyn et al., 2014; Gajardo et al., 2016). These microbes aid nutrient absorption and food breakdown (Moore et al., 2006; Wang AR, et al., 2018).

Surgeonfish are herbivorous, inhabiting warm tropical seas and feeding primarily on algae. Their gut microbiota includes *Acinetobacter, Arcobacter, Fusobacterium, Vibrio*, and *Photobacterium* (Miyake et al., 2016; Ngugi et al., 2017; Parata et al., 2020), with specialized metabolic capacities for degrading algal polysaccharides, essential for their digestion and nutrient acquisition (**Table 1**).

### 2.3 Shellfish gut microbiota

The expansion of commercial shellfish aquaculture, particularly shrimp farming, has coincided with a rise in infectious as well as non-infectious disease etiologies. High prevalence has been observed for filamentous bacteria, peritrich protozoans, invasive bacterial species, and fungi. However, viral agents constitute a critical category of pathogenic microorganisms, having achieved widespread dissemination within shrimp aquaculture facilities. Initially geographically restricted within wild shrimp populations, these viruses have attained global distribution primarily due to the translocation of broodstock and post-larval stages from hatchery systems to geographically distinct locations. A survey by the Global Aquaculture Alliance (GAA) indicated that 60% of shrimp farming losses are attributed to viral infections, while bacterial infections account for 20% of these losses. The first major microbial disease epizootic in shrimp aquaculture occurred in Taiwan during the 1980s, with Monodon baculovirus (MBV) identified as the etiological agent (Flegel et al., 2008). Following this event, outbreaks of infectious hypodermal and hematopoietic necrosis virus (IHHNV) were reported in the United States (Lightner, 1996), yellow head virus (YHV) in Thailand (Flegel, 1997), and Taura syndrome virus (TSV), also in the United States (Brock, 1998). The period from 1993 to 2003 presented further challenges to the shrimp aquaculture sector, characterized by the extensive epizootic of white spot syndrome virus (WSSV), initially documented in China in 1992, which subsequently exhibited rapid pan-Asian spread, resulting in significant economic losses (Flegel et al., 2008). The study documented the presence of six microbial diseases affecting the shrimp *Litopenaeus vannamei* within Indian aquaculture systems (Gunalan et al., 2014). These included black gill disease, Taura Syndrome Virus (TSV), Infectious Hypodermal and Hematopoietic Necrosis Virus (IHHNV), white muscle disease (WMD), white gut disease, and muscle cramp disease. While bacterial etiologies, particularly those involving *Vibrio* spp., have a long-established association with shrimp health and are often correlated with compromised physiological status or suboptimal aquaculture management practices, even immunocompetent individuals can be susceptible to infection under environmental conditions conducive to pathogenic proliferation. The primary sites of bacterial infection are frequently the branchiae and alimentary canal. In severe cases, filamentous bacteria can colonize the branchial lamellae (Johnson et al., 1995). A diverse array of over 20 bacterial species, encompassing human pathogens such as *Vibrio cholerae*, *V. parahaemolyticus*, and *V. vulnificus*, alongside aquatic pathogens like *V. harveyi* and *V. penaeicida*, have been implicated in significant shrimp disease outbreaks (Otta et al., 2001). Notably, *Vibrio harveyi* has been associated with shrimp mortality events and is recognized as the causative agent of brown gill syndrome in *Penaeus monodon* (Karunasagar et al., 1994; Flegel and Pasharawipas, 1998). Filamentous bacteria, including *Leucothrix mucor*, *Thiothrix* sp., *Flexibacter* sp., *Flavobacterium*, and *Cytophaga* sp., have been observed to infect shrimp, particularly during larval ontogeny, manifesting in clinical signs such as branchial discoloration, reduced growth rates, and increased mortality (Karunasagar et al., 2007).

Shrimp farming involving penaeid species faces vulnerability to a wide spectrum of viral pathogens, with over twenty identified as the causative agents of various disease conditions. These viruses are classified within several families, encompassing Parvoviridae, Baculoviridae, Picornaviridae, and Toga-like viruses. The World Organisation for Animal Health (OIE) has identified seven viral pathogens as critically important due to their effects on shrimp aquaculture (Dehaumont, 2004). The alimentary canal of crustaceans, particularly the posterior intestine, offers a favorable milieu for a substantial community of microorganisms (Ceccaldi, 1997). This microbiota is involved in diverse physiological functions, including aiding in nutrient breakdown, synthesizing digestive enzymes, and supplying essential micronutrients such as vitamins (Ceccaldi, 1997). Comparative investigations of shrimp intestinal microbiota across different environmental conditions have indicated variations in microbial composition. Oetama et al. (2016) showed that Proteobacteria represents the dominant bacterial phylum in shrimp originating from both contaminated and less contaminated aquatic environments, followed by less prevalent phyla such as Bacteroidetes, Fusobacteria, and Firmicutes. Of particular note, potentially disease-causing bacteria belonging to the orders Vibrionales and Pseudoaltermonadales were also identified.

Studies examining the intestinal microbial communities of *Litopenaeus vannamei* have characterized a diverse array of bacteria, exceeding 100 distinct isolates. Within these communities, the genera *Photobacterium*, *Vibrio*, *Aeromonas*, *Xanthomonas*, *Agrobacterium*, and *Bacillus* constitute the predominant taxa (Li et al., 2018). Although the phylum Proteobacteria is generally recognized as the dominant and putatively advantageous component of the healthy shrimp gut microbiota, potentially pathogenic bacteria, including *Pseudomonas*, *Flavobacterium*, *Escherichia*, *Aeromonas*, *Vibrio*, *Rickettsia*, *Shewanella*, and *Desulfovibrio*, are also detected at lower relative abundances (Cardona et al., 2016; Qiao et al., 2017).

Research has extensively investigated the modulation of shrimp gastrointestinal microbiota via prebiotic and probiotic administration to influence health outcomes. Numerous studies have assessed their capacity to bolster immune competence and alter the gut microbial profiles (Li et al., 2018; Yukgehnaish et al., 2020). Gut microbial dysbiosis has been implicated in the etiology of various shrimp pathologies. For instance, acute hepatopancreatic necrosis disease (AHPND) in *Litopenaeus vannamei* is associated with a significant diminution in bacterial richness in affected individuals compared to healthy conspecifics (Liu et al., 2018). Similarly, White Spot Syndrome Virus (WSSV) infection induces a shift in microbiota composition, characterized by an augmentation of Proteobacteria and Fusobacteria and a reduction in Bacteroidetes and Tenericutes (Wang C, et al., 2018). Notably, Huang and Guo reported that microbiota richness in WSSV-infected shrimp showed no significant changes when cultured in biofloc systems, suggesting that this approach may not confer resistance to WSSV (Huang et al., 2018; Guo et al., 2020). White faeces syndrome (WFS) has been correlated with modifications in the gut microbiota, specifically an increase in Tenericutes and Firmicutes and a decrease in Proteobacteria in diseased shrimp (Hou et al., 2018). Investigations into cotton shrimp-like disease (CSL) have indicated an elevation in *Tenacibaculum* bacteria, although the overall microbiota composition of CSL-affected shrimp demonstrated similarity to that of healthy shrimp (Zhou et al., 2019). Furthermore, Liang et al. (2020) documented significant variations in the microbiota composition of shrimp afflicted with blue body syndrome (BBS) compared to healthy shrimp however, further research is warranted to fully elucidate these relationships. Studies on the microbiota of oysters (*Crassostrea virginica* and *Crassostrea gigas*) have concentrated on their interactions with bacteria, including human pathogens such as *Vibrio parahaemolyticus* and *V. vulnificus* (Johnson et al., 2010; Sobrinho et al., 2010). Research by King et al. (2012) utilizing high-throughput sequencing methodologies revealed a predominance of Mollicutes and Planctomyctes in the oyster stomach microbiota, whereas the intestinal microbiota exhibited distinct microbial assemblages. These findings indicate the presence of novel microbial communities within oyster digestive systems, although the functional roles of these microbiotas largely remain to be determined (**Table 2**).

**Table 2:** Gut microbiota composition and function in shellfish.

Species	Feeding Behaviour	Habitat	Predominant microbiota	Key Genera	Microbial Roles	Cited Studies
Shrimp *(Penaeus monodon)*	Carnivorous/Omnivorous	Aquaculture ponds	Proteobacteria, Bacteroidetes, Fusobacteria, Firmicutes	*Vibrio, Pseudomonas, Aeromonas, Flavobacterium, Shewanella*	Digestive processes, immune modulation, pathogenicity	Oetama et al., 2016; Li et al., 2018; Otta et al., 1999; Flegel et al., 2008
Shrimp *(L. vannamei*)	Omnivorous	Aquaculture systems	Proteobacteria, Firmicutes, Fusobacteria	*Vibrio, Pseudomonas, Flavobacterium, Aeromonas, Enterobacteriacea*	Gut health, disease modulation, pathogen protection, impacts on shrimp growth and disease like WSSV and AHPND	Li et al., 2018; Liu et al., 2018; Qiao et al., 2017; Wang et al., 2019; Rajeev et al., 2021
Shrimp (*P. monodon)*	Carnivorous	Marine coastal systems	Proteobacteria, Firmicutes	*Vibrio harveyi, Leucothrix mucor, Flexibacter, Aeromonas*	Pathogen invasion in gill filaments, digestive tract infections, mass mortalities	Karunasagar et al., 2007; Otta et al., 2001; Johnson et al., 1995
Oysters (*C. virginica)*	Filter feeder	Marine, coastal habitats	Proteobacteria, Firmicutes, Mollicutes, Planctomyces	*Vibrio parahemolyticus, Cristispira, Stappia*	Symbiotic relationship in the digestive system, pathogen interaction	King et al., 2012; Romero et al., 2002; Mayasich & Smucker 1987; Boettcher et al., 2005
Oysters (*C. gigas)*	Filter feeder	Marine, coastal habitats	Proteobacteria, Firmicutes	*Vibrio, Arcobacter, Cristispira, Stappia*	Gut health, pathogen interaction	Faury et al., 2004; Hernández-Zárate & Olmos-Soto, 2006; Johnson et al., 2010
Pearl oyster	(*Pinctada fucata martensii*)	Marine, coastal habitats (inner bay vs. open sea)	38 phyla including Proteobacteria, Tenericutes, Cyanobacteria, Planctomycetes; 272 genera	*Core OTUs: Tenericutes* spp.*, Cyanobacteria* spp.*, Planctomycetes* spp.	Cofactor and vitamin metabolism, carbohydrate and amino acid metabolism, lipid metabolism, adaptation to environmental microbiota	Zheng et al., 2021
Pacific white shrimp	(*Litopenaeus vannamei*)	Aquaculture systems	Proteobacteria, Firmicutes, Bacteroidetes, Fusibacter with KO diet)	*Fusibacter, Photobacterium, Vibrio*	Antioxidant activity, innate immunity enhancement, lipid & terpenoid/polyketide metabolism; Fusibacter positively correlated with immune and antioxidant parameters	Liang et al., 2022
Chinese mitten crab	(*Eriocheir sinensis*)	Freshwater aquaculture	GH: unclassified_c_Alphaproteobacteria; RH: *Flavobacteria bacterium BAL38*, *Paraburkholderia ferrariae*, *Legionella* sp.	*Shewanella* sp. *MR-7 positively correlated with key differential metabolites*	Amino acid and lipid metabolism, pigment production, protein digestion and absorption, aminoacyl-tRNA biosynthesis	Zhu et al., 2023

Cross-ecosystem comparisons reveal that dietary ecology and environment are the primary determinants of microbiota structure and function. Freshwater herbivores depend on cellulolytic bacteria for fibre degradation, while carnivorous freshwater and marine fish harbor lipid and protein-degrading microbes, and marine herbivores rely on polysaccharides for algal digestion. In contrast, shellfish exhibit complex host microbe pathogen dynamics, heavily influenced by aquaculture practices and viral disease pressures. Across all groups, the gut microbiota consistently supports three recurring functional themes, nutrient assimilation through the breakdown of carbohydrates, proteins, and lipids, immune modulation and pathogen resistance through commensal protection; and host development and reproduction through microbiota mediated growth, maturation, and sex differentiation. Collectively, these findings underscore that gut microbiota are not passive residents but active contributors to the health, productivity, and resilience of fish and shellfish.

## 3 Functional roles of fish and shellfish gut microbiota in nutrition and metabolism

**Figure 2:**
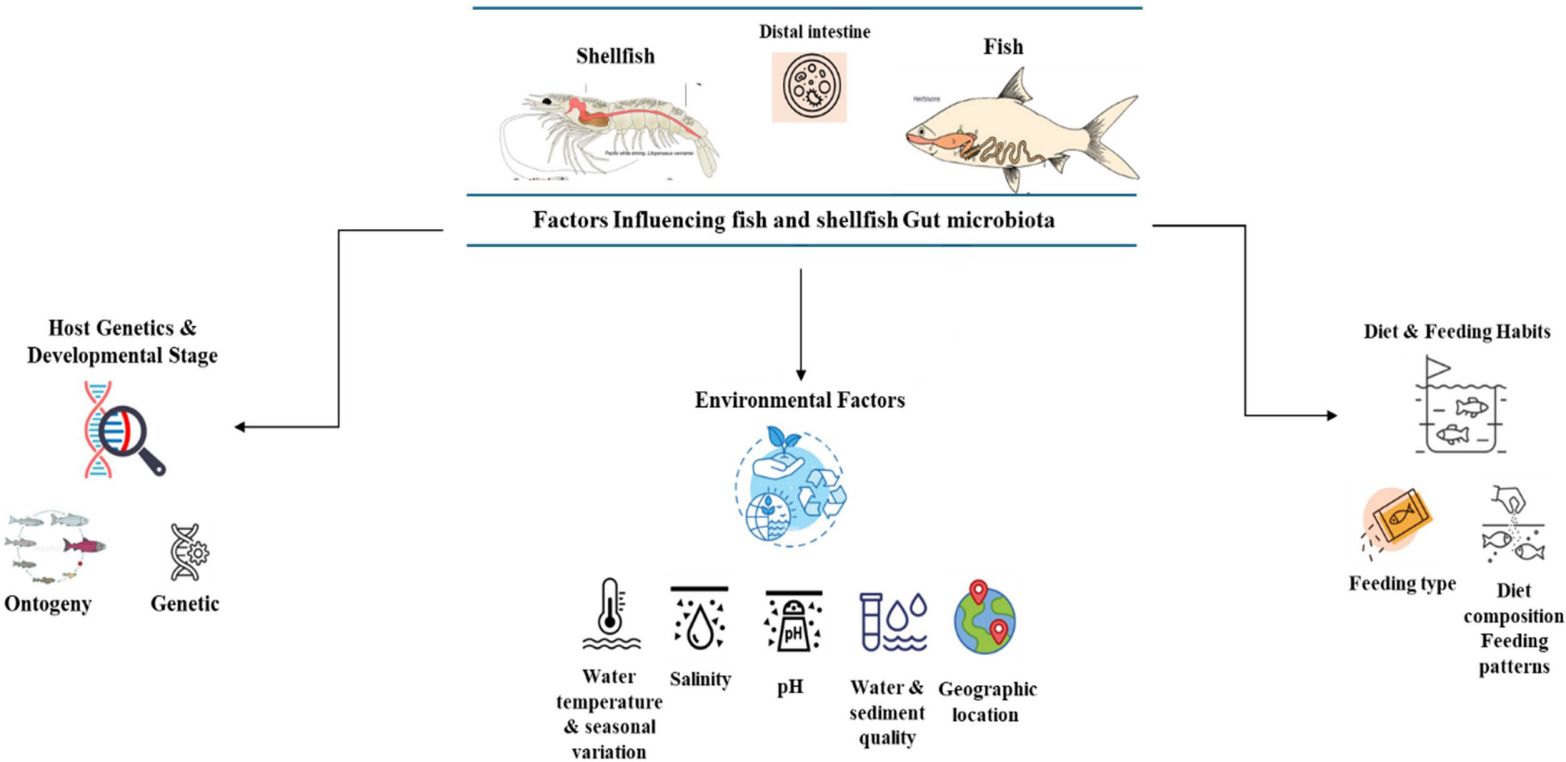
Mechanistic roles of gut microbiota in fish and shellfish health.

The intestinal microbial community significantly influences the nutritional physiology and metabolic homeostasis of fish by actively contributing to the breakdown and absorption of food constituents and modulating the host’s metabolic processes (**Figure 2**).

Fish rely on their gut microbes to enzymatically digest complex parts of their diet. Numerous bacterial species within the fish gut produce digestive enzymes, such as amylases for carbohydrate breakdown, proteases for protein hydrolysis, and lipases for lipid catabolism, which augment or compensate for the host’s own enzymatic capabilities (Ray et al., 2022; Zhang et al., 2024). This microbial enzymatic activity significantly enhances the efficiency of nutrient digestion in the host (Zhang et al., 2024). Furthermore, the gut microbiota stimulates the proliferation and maturation of the intestinal epithelium, thereby increasing the absorptive surface area available for nutrient uptake (Nayak, 2010). Zebrafish research indicates that gut bacteria promote an elevated count and size of lipid droplets within intestinal epithelial cells, indicating improved lipid absorption (Semova et al., 2012). Cheesman et al. (2011) further elucidated that the microbiota, in conjunction with Wnt signalling pathways, promotes intestinal epithelial cell proliferation by stabilizing β-catenin within gut tissues, potentially contributing to enhanced digestive capacity. Inherent metabolic pathways in fish often demonstrate suboptimal efficiency in processing carbohydrates. However, gut-associated microorganisms enhance carbohydrate digestion through the production of relevant enzymes and by potentiating the activity of the host’s digestive enzymes (Zhang et al., 2024). The microbial fermentation of carbohydrates yields short-chain fatty acids (SCFAs), which are subsequently absorbed by the host and utilized as energy substrates (Pardesi et al., 2022; Hao et al., 2017; Petit et al., 2022). *Cetobacterium somerae*, a dominant bacterial taxon in the gut of many freshwater fish species, has been shown to improve glucose homeostasis via the production of acetate, which exerts its effects through the activation of parasympathetic pathways in zebrafish (Wang J, et al., 2021). Similarly, the administration of *Bacillus amyloliquefaciens* SS1 in Nile tilapia fed a high-carbohydrate diet resulted in improved metabolic phenotypes, including reduced fasting glucose levels and decreased lipid accumulation, potentially mediated by an increase in acetate-producing bacteria (Petit et al., 2022). A comparative analysis of the gut microbiota in herbivorous and carnivorous fish by Liu et al. (2016) revealed that cellulase and amylase activities were more closely associated with herbivores, whereas trypsin activity correlated with carnivores. It seems the specific makeup of the microbial community adjusts to the host’s diet and actively participates in breaking down and using carbohydrates and proteins.

The intestinal microbiota significantly enhances host energy extraction via multiple pathways, encompassing the modulation of lipid absorption and the transformation of bile acid profiles and the adjustment of genes involved in maintaining energy balance, Guo and colleagues in 2017 showed that zebrafish consuming diets rich in nucleotides displayed lower basal metabolic rates as a result of changes in their gut bacteria, leading to improved energy storage and growth (Guo et al., 2017). Similarly, Zhang studied that *Citrobacter* bacteria isolated from Nile tilapia intestines enhanced energy extraction in fish consuming a high-fat diet, highlighting the microbiota’s ability to modulate the adverse outcomes associated with suboptimal nutrient intake (Zhang et al., 2020). Furthermore, De La Torre Canny et al., 2021 indicated that *Plesiomonas* species reduced fat accumulation in zebrafish, suggesting that specific gut microbes can counteract the obesogenic effects of environmental contaminants like tributyltin (TBT). Bile acids, crucial for the digestion and absorption of lipids, undergo microbial transformation in the gut into secondary bile acids that influence glucose and lipid metabolism. In zebrafish, the primary biliary acids, 5α-cyprinol sulfate (5αCS) and taurocholic acid (TCA), undergo biotransformation mediated by the gut microbiota (Wen et al., 2021). Specifically, an identified Acinetobacter species exhibited the capacity for TCA deconjugation, potentially leading to the activation of farnesoid X receptor (FXR) signaling, a key regulatory axis in lipid and energy homeostasis.

Xia et al. (2023) also observed that bile acids enhance intestinal barrier function through both direct mechanisms and microbiota-dependent routes, potentially impacting nutrient assimilation and metabolic processes. The feed conversion ratio (FCR) represents a crucial economic parameter in aquaculture. Gut microbiota contributes to enhanced FCR by improving digestive and metabolic efficiency. Although more extensively investigated in monogastric animals, accumulating evidence from fish also supports this notion. Specific probiotic strains, such as *Lactobacillus acidophilus* (Singh et al., 2014), *Bacillus coagulans* (Adeshina et al., 2020), and *Acinetobacter* (Amoah et al., 2019), have been associated with improved FCR in fish, although the underlying mechanisms remain to be fully elucidated. Dvergedal et al. (2020) identified three operational taxonomic units (OTUs) that exhibited a positive correlation with enhanced feed efficiency and carbon metabolism in *Salmo salar*. Similarly, Bozzi et al. (2021) reported a positive association between *Mycoplasma* abundance and both physiological condition and somatic weight in salmon, indicating a beneficial microbial role in growth and energy utilization. Intestinal microbes can modulate host gene expression related to metabolic pathways. For example, studies in *Danio rerio* have demonstrated microbiota-mediated regulation of genes involved in lipid uptake, fatty acid metabolism, and energy storage (Sheng et al., 2018). In zebrafish, Semova and colleagues demonstrated in 2012 that the gut microbial community enhanced the uptake of lipids and the accumulation of lipid droplets within the intestinal and hepatic tissues. This observation suggests that the microbially mediated influence on host metabolic pathways particularly lipid processing may represent a conserved biological mechanism across vertebrate species.

The intestinal microbiota of crustaceans, notably shrimp, is fundamental to their nutritional physiology, metabolic homeostasis, immune system function, and capacity to withstand environmental perturbations. Considering the limited capacity of shrimp to digest complex food components such as carbohydrates, proteins, and fats, the gut microbiota plays a vital role in completing these metabolic processes. The dynamic nature of this microbial community’s structure and function allows for rapid adjustments in response to dietary changes, environmental factors, and stressors, with downstream effects on host health and productivity. Shrimp is critically dependent on their gut microbiota for the assimilation of complex feed components. The intestinal microbiota metabolizes unabsorbed nutrients and generates diverse metabolites that positively influence the host digestive processes and general health (Qiao et al., 2017). These micro-organisms express enzymes including proteases, lipases, and amylases that contribute to the catabolism of proteins, lipids, and carbohydrates, respectively. Distinct anatomical sections of the shrimp gastrointestinal tract exhibit specialized microbial metabolic roles. The foregut microbiota is predominantly involved in the biotransformation of amino acids and carbohydrates. The community of microorganisms residing in the insect midgut is crucial for processing fats, polyketide compounds, and terpenoids. The hindgut microbiota is more engaged in vitamin biosynthesis, energy production, and cofactor metabolism (Garibay-Valdez et al., 2021).

The evident functional diversification highlights the adaptive specialization of the gut microbiota, driven by the spatial heterogeneity and nutrient gradients along the host’s digestive tract. Dietary carbohydrates function not only as a principal energy substrate for shrimp but also as crucial substrates supporting the metabolic activities of the gut microbial community. Through fermentative pathways, these microorganisms metabolize carbohydrates, yielding short-chain fatty acids (SCFAs) and other biologically active compounds. The inclusion of various carbohydrates, such as glucose, sucrose, xylooligosaccharides, and starch, in the dietary formulations for shrimp has been shown to positively influence the composition and resilience of their gut microbial communities. This modulation of the intestinal microbiota is associated with enhanced gut health and improved overall growth performance in shrimp aquaculture (Chen et al., 2021; Gyan et al., 2022). Furthermore, modulation of the carbon-to-nitrogen (C/N) ratio in feed has been shown to optimize the gut microbial architecture, increasing microbial efficiency in nutrient catabolism and anabolism, thereby enhancing shrimp productivity (Guo et al., 2020). An optimized C/N ratio fosters the proliferation of beneficial bacterial taxa and supports a conducive environment for microbial fermentation and energy acquisition. Gut microbiota-mediated protein and lipid metabolism is critical for maximizing feed utilization efficiency in shrimp. Modifications in the dietary protein composition or ratio induce shifts in the gut microbial community towards species exhibiting enhanced proteolytic capabilities, consequently improving the digestion and assimilation of amino acids (Gyan et al., 2022).

The nature of dietary lipid sources, such as soybean oil, tallow, or linseed oil, exerts a considerable impact on the structural assembly of the intestinal microbial community. These alterations in microbial composition modulate lipid metabolic pathways and correlate with observed differences in immunological responses and growth performance (Zhang et al., 2014). Furthermore, specific microbial taxa contribute to the biotransformation of bile acids, indirectly facilitating the processes of lipid emulsification and subsequent absorption. The community of microorganisms residing in the shrimp’s gut is essential for regulating its immune system. A balanced and diverse microbial ecosystem can confer protection against pathogenic organisms through mechanisms including competitive exclusion, the enhancement of mucosal immunity, and the production of antimicrobial compounds. Following infection with White Spot Syndrome Virus (WSSV), shrimp demonstrate shifts in their core gut microbial profile. These microbial dysbiosis events are frequently associated with an increased production of antiviral metabolites of microbial origin, which contribute to the maintenance of immune homeostasis and provide defence against the disease (Zhang and Sun, 2022).

The increasing acknowledgement of probiotic supplementation as a more environmentally sustainable and safe approach compared to antibiotic administration in shrimp aquaculture for disease control is significant. Probiotic bacteria, including genera such as *Lactobacillus* and *Bacillus*, have demonstrated positive influences on shrimp growth metrics, immune competence, and survivability. These beneficial effects are mediated through the promotion of advantageous microbial communities and the attenuation of pathogenic organism burdens (Holt et al., 2020). Environmental variables, with a particular emphasis on salinity, significantly impact the compositional architecture of the shrimp gastrointestinal microbiota. Research on *Penaeus monodon* indicated that shrimp exposed to an initial acclimatization at 20 ppt salinity, followed by transfer to environments of 10 ppt and 30 ppt, exhibited modifications in microbial community structure. Specifically, the relative abundance of *Vibrio* species established sensitivity to salinity shifts (Chaiyapechara et al., 2012). While *Vibrio* populations increased in correlation with elevated salinity levels, the relative abundance of other genera, remained relatively constant, indicating a degree of stability within the core microbiota.

Alterations in environmental salinity induce shifts in the microbial community structure, which are mirrored at the molecular level. Metatranscriptomic studies show that genes related to stress response and immunity are expressed at varying levels, underscoring the interconnected impact of environmental salinity on the transcriptional profiles of both the host organism and its associated microbiota. However, the precise nature of this interaction whether the shrimp actively regulate these microbial shifts or passively respond to them requires further investigation. Nutrient deprivation, specifically starvation, elicits substantial modifications in the gut microbiota composition and functional attributes. In shrimp experiencing starvation, a downregulation of digestive enzyme activity is observed concurrently with an upregulation of immune response-related genes. Functional pathway analysis reveals a diminished capacity for carbohydrate, protein, lipid, glycan, and enzyme metabolism under conditions of nutrient scarcity (Dai et al., 2020). These physiological alterations heighten the host’s susceptibility to pathogenic invasion, emphasizing the importance of the gut microbiota in maintaining metabolic and immunological homeostasis during periods of stress. Contemporary research has begun to elucidate correlations between the compositional characteristics of the gut microbiota and parameters of shrimp breeding performance. Specific microbial signatures have been linked to enhanced growth rates and weight gain, indicating the potential utility of gut microbial structure as a biomarker for selective breeding initiatives in shrimp aquaculture (Fan & Li, 2019). These findings lend support to the integration of microbiota analysis into aquaculture breeding programs as a strategy to improve sustainability and overall productivity.

## 4 Sequencing strategies for gut microbiota analysis in fish and shellfish

Advances in next-generation sequencing (NGS) have greatly enhanced the characterization of fish and shellfish gut microbiota, primarily through targeted amplicon sequencing and shotgun metagenomics (Wensel et al., 2022). While differing in scope and resolution, both approaches have transformed microbiome research in aquaculture.

Amplicon sequencing, typically targeting the 16S rRNA gene for bacteria or ITS regions for fungi, offers cost-effective and scalable profiling of microbial communities (Yang et al., 2025). It has been widely applied to assess how gut microbiota shift across diets, developmental stages, and environmental or health conditions (Perez-Bustamante et al., 2024). Although this strategy cannot directly resolve functional genes or capture non-bacterial taxa, it remains well suited for large-scale ecological comparisons.

Shotgun metagenomics, which sequences total genomic DNA without prior amplification, enables comprehensive profiling of both microbial composition and functional capacity (Yang et al., 2025). This approach achieves strain-level resolution across bacteria, archaea, viruses, and microeukaryotes (Talwar et al., 2018), and allows annotation of metabolic pathways, virulence factors, antimicrobial resistance genes, and mobile elements. Such functional insights are particularly valuable for elucidating microbial contributions to nutrient assimilation, immune modulation, pathogen defense, and stress tolerance in aquaculture species (Khurana et al., 2020). For instance, shotgun studies have linked microbial shifts to dietary efficiency, probiotic action, and disease resistance, thereby informing management strategies that enhance productivity and sustainability.

The choice of method depends on study objectives: amplicon sequencing is ideal for high-throughput surveys of microbial dynamics across treatments or time points (Spilsbury et al., 2022), whereas shotgun metagenomics is preferred for mechanistic analyses of host–microbe interactions and functional profiling (Yang et al., 2025). Increasingly, combined approaches are employed to leverage the breadth of amplicon surveys with the depth of shotgun datasets, providing a more holistic understanding of gut microbial ecology (Sheth et al., 2016).

Looking forward, integration of metagenomics with transcriptomics, metabolomics, and metaproteomic will be critical for linking microbial identity with activity. These multi-omics strategies are beginning to reveal not only community composition but also metabolic function within the gut, creating new opportunities to connect microbiome dynamics with growth performance, disease resistance, and sustainability outcomes in aquaculture.

## 5 Factors influencing the composition of fish and shellfish gut microbiota

**Figure 3:**
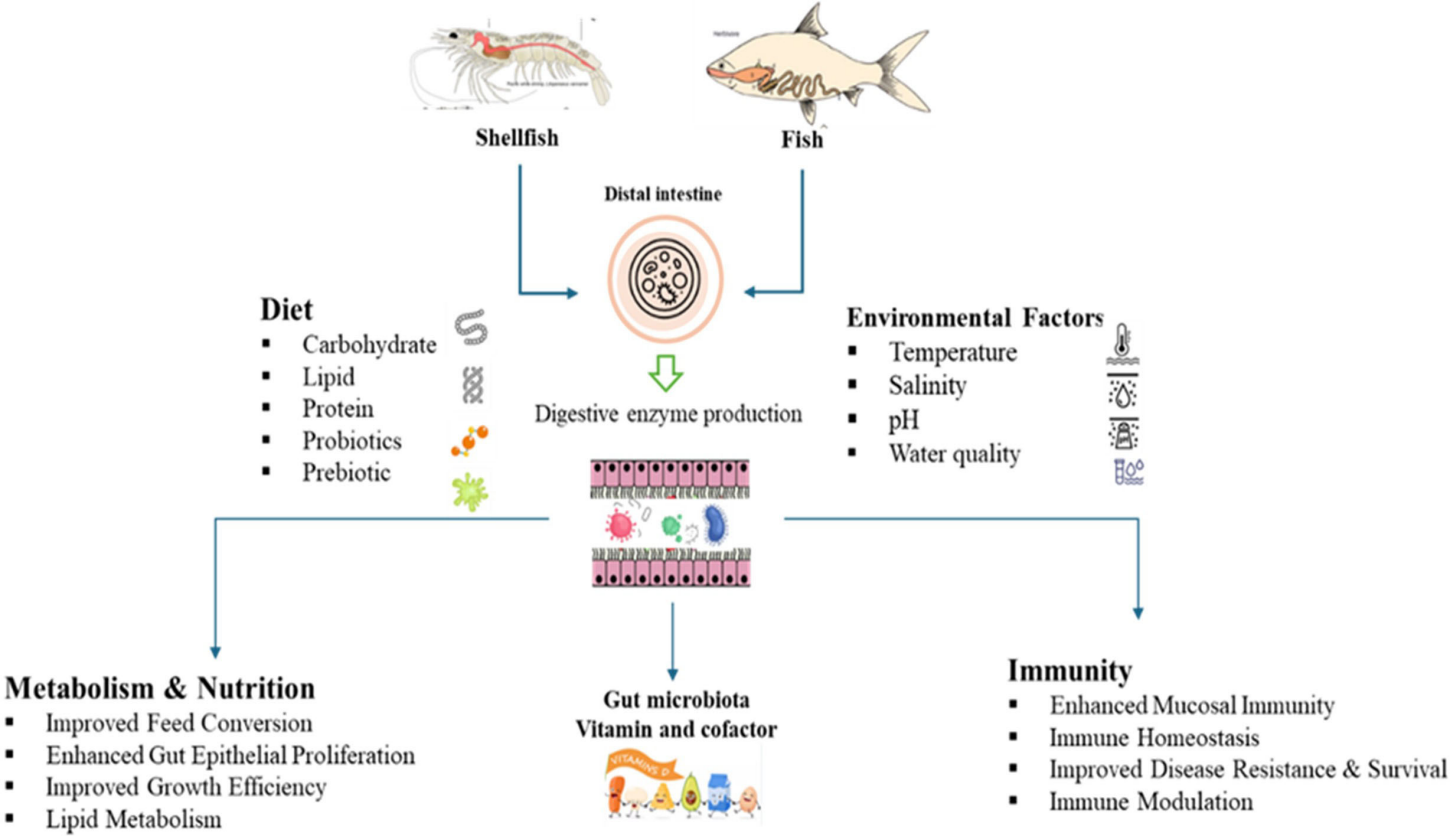
Factors influencing fish and shellfish gut microbiota.

The structure and variety of microorganisms inhabiting the fish gut are determined by a complex and ever-changing combination of environmental conditions, inherent biological traits, and nutritional inputs. These factors exert specific effects that vary across the life cycle of the fish and in different habitats. An increasing amount of scientific evidence indicates that comprehending these intricate relationships is essential for refining fish farming techniques, as well as for maintaining fish well-being, strengthening their defense mechanisms, and fostering ecological adaptability (Diwan et al., 2023a; Ou et al., 2021) (**Figure 3**).

### 5.1 Environmental factors

#### 5.1.1 Water temperature and seasonal variation

Fish gut microbiota composition is strongly affected by temperature, driving the proliferation or decline of specific microbial taxa in response to thermal conditions (Ghosh et al., 2022; Hassenrück et al., 2020; Sepúlveda-Quiroz et al, 2021). In young milkfish (*Chanos chanos*), alterations in ambient temperature between 26 and 33 °C elicited notable modifications in the intestinal microbiota composition, thereby supporting the host’s physiological acclimatization to variable thermal conditions (Hassenrück et al., 2020). In a parallel observation within *Oncorhynchus tshawytscha*, differing temperature regimes induced a shift in the dominant microbial communities, characterized by the displacement of families like Vibrionaceae and their subsequent substitution with Fusobacteriaceae and Brevenemataceae (Steiner et al., 2022; Dulski et al., 2020; Hovda et al., 2012).

#### 5.1.2 Salinity

Salinity represents a pivotal environmental determinant shaping the gut microbial architecture, particularly for species undergoing transitions between marine and freshwater habitats. In *Salmo salar*, the migration from saltwater to freshwater ecosystems induced alterations in the prevailing bacterial phyla. Notably, the genera *Escherichia* and *Shigella* exhibited increased relative abundance in populations inhabiting seawater (Morales et al., 2022). Conversely, certain taxa, including the phylum Proteobacteria and the genus *Lactobacillus*, demonstrated a capacity to withstand fluctuations in salinity in aquaculture settings involving *Oncorhynchus tschawytscha* (Zhao et al., 2020; Liu et al., 2023).

#### 5.1.3 pH

The acidity of aquatic habitats significantly impacts the equilibrium of gut microorganisms. Deviations from a neutral pH, whether towards acidic or alkaline conditions, can disrupt this microbial balance (homeostasis) and favor the proliferation of disease-causing microbes (pathogens). Fonseca et al. (2019) observed a decrease in beneficial lactic acid bacteria in sea bream when pH was lowered. Conversely, Kong et al. (2022) and Shang et al. (2024) describes an increase in detrimental *Vibrio* species in common carp under alkaline conditions. Exposure of the Pacific oyster, *Crassostrea gigas*, and other consumable oyster species to conditions of ocean acidification, characterized by decreased pH levels, has been observed to induce shifts in their associated microbial communities (Kong et al., 2022).

#### 5.1.4 Water and sediment quality

Aquatic microbial communities, residing in the water column and sediment, exert a considerable influence on fish gut microbiota via ongoing exposure. In shrimp species like *Litopenaeus vannamei* and *Penaeus japonicus*, studies have evidenced a notable similarity between the microbial compositions of the ambient water and the host’s intestinal tract (Huang et al., 2018; Song et al., 2020). These environmental microbial assemblages function as microbial reservoirs, facilitating the ingestion and subsequent colonization of the gut by microorganisms (Huang et al., 2021).

#### 5.1.5 Geographic location

Geographic partitioning results in environmental heterogeneity, characterized by variations in salinity, temperature, and the spectrum of microorganisms. These variations, in turn, influence the structural makeup of the gut microbial communities within organisms. For instance, research employing high throughput 16S rRNA sequencing by Liu et al. (2022b); Liu et al. (2022a) investigations revealed significant variations in the gut microbial community structure across *Megalobrama terminalis* populations residing in the Pearl, Moyang, and Wanquan River systems. This phenotypic divergence is attributed to allopatric speciation driven by genetic drift and adaptation to distinct environmental pressures. Conversely, studies by Noman et al. (2024) indicated a negligible impact of geographic location on the gut microbiota of gilthead seabream (*Sparus aurata*) and European seabass (*Dicentrarchus labrax*), suggesting that certain species may maintain relatively stable microbial communities irrespective of their geographic distribution.

### 5.2 Host genetics and developmental stage

Genetic traits inherited from a host significantly impact the development of their microbial communities, the way their immune system functions, and their enzyme production, ultimately determining the makeup of the gut microbiota (Naya-Català et al., 2022). Studies in *Gasterosteus aculeatus* revealed that genetically differentiated populations harbored divergent gut microbial assemblages (Smith et al., 2015). Conversely, in *Ictalurus punctatus* and *Ictalurus furcatus*, environmental variables appeared to be the dominant drivers of gut microbial composition when ontogenetic trajectories were comparable (Bledsoe et al., 2018). Furthermore, the host’s developmental phase represents a crucial determinant of microbial community structure, the microbiota undergoes temporal shifts in relation to host maturation., *Sparus aurata* in later life stages exhibited greater microbial richness compared to younger individuals (Parata et al., 2020). Analogous ontogenetic patterns of microbial succession have been documented in Acipenseridae species and *Silurus meridionalis*, where microbial community dynamics correlated with host developmental transitions (Parata et al., 2020).

### 5.3 Diet and feeding habits

The interplay between piscian alimentary regimes and foraging habits significantly modulates the constitution of their intestinal microbial consortia. Herbivorous generally possess comparatively longer alimentary canals and harbor distinct gut microbial communities compared to carnivorous species. The impact of nutritional factors on the composition and function of these microbial consortia is well-established in scientific publications, for instance, in Atlantic salmon, diets rich in carbohydrates have been shown to diminish overall bacterial load while fostering the proliferation of taxa specialized in carbohydrate metabolism (Villasante et al., 2019). Ontogenetic shifts in feeding patterns, as observed in *Megalobrama amblycephala*, result in microbial community reorganization and modifications in enzymatic activities (Wei et al., 2018). Furthermore, periods of nutritional deprivation also exert influence on gut microbial assemblages; in *Plectropomus leopardus*, Firmicutes was the predominant phylum during periods of feeding, whereas Proteobacteria became dominant during fasting (Mekuchi et al., 2018).

## 6 Conclusion

Gut-associated microbial communities in fish and shellfish are now widely regarded as a functional “accessory organ” that exerts significant influence on host physiology, nutrition, and metabolic efficiency. These microbial assemblages differ considerably among freshwater and marine species, as well as across various groups of shellfish, reflecting the combined effects of host traits, ecological roles, and environmental conditions. Through processes such as enzymatic degradation of complex substrates, vitamin biosynthesis, and enhancement of nutrient absorption, the gut microbiota directly contributes to host growth and health. The composition of these microbial communities is not static but dynamically shaped by multiple internal and external drivers, including environmental parameters (temperature, salinity, pH, sediment quality, and geography) and host-related factors (genetics, development, and diet). Collectively, this host microbe environment interaction forms a regulatory network essential for maintaining intestinal balance and overall organismal health. Despite notable advances, the precise cellular, molecular, and metabolic mechanisms that govern these interactions remain insufficiently understood. Future research should focus on unravelling the influence of gut microbiota on host gene expression, immune regulation, and metabolic pathways, using integrative multi-omics and controlled experimental approaches. In addition, the microbial communities of aquatic organisms harbor an untapped reservoir of bioactive molecules with promising applications in pharmaceuticals, nutraceuticals, and sustainable aquaculture practices. A more comprehensive mechanistic understanding will not only optimize health and productivity in aquaculture but also open new frontiers in microbial biotechnology and environmentally responsible cultivation of fish and shellfish.

## 7 Future perspectives and recommendation

To significantly propel gut microbiota research forward, foundational experimental methodologies necessitate enhancement. The integration of sophisticated multi-omics analyses offers deeper understanding into the gut microbiota’s role, function, and composition, facilitating the optimization of the intestinal ecosystem for future applications (Ou et al., 2021). Gnotobiotic piscine models, such as zebrafish and stickleback, present valuable systems for these investigations (Zhang et al., 2021). Diverse bioengineering strategies can be employed to cultivate a beneficial microbiota or to remediate dysbiosis in animal models. However, a thorough understanding of the interplay between host disease states and the intestinal microbial community is crucial for effective implementation (Filardo et al., 2024). A synergistic application of various engineering methodologies, targeted at gut microbiota modulation, represents a primary focus for future research endeavours (Nath et al., 2022). Expanding beyond conventional engineering approaches, metabolic engineering strategies hold promises for improved outcomes in gut microbiota modulation (Li et al., 2023). CRISPR-based technologies offer the potential to significantly advance engineering techniques with enhanced precision and efficiency (Careaga, 2024). Beyond single-strain manipulation, the application of synthetic microbial communities (SynComs) provides an emerging strategy. These rationally designed consortia can deliver consistent functions such as nutrient assimilation, pathogen resistance, and stress tolerance, thereby enabling more predictable and stable outcomes in aquaculture systems. Another promising direction is precision aquaculture microbiome engineering, where microbial interventions are tailored to host species, developmental stage, or environmental conditions. Supported by artificial intelligence (AI) and machine learning, such targeted approaches could optimize microbial community dynamics in real time, thereby improving feed conversion efficiency, growth, and disease resilience. In addition, integrating microbiome engineering with climate change resilience strategies represents a crucial future perspective. Microbial communities may buffer fish and shellfish against stressors such as ocean acidification, salinity fluctuations, and rising water temperatures. Designing microbiomes with adaptive traits could thus strengthen the robustness of aquaculture systems under global climate variability.

The development of natural bacteria-derived therapeutics for various human diseases represents a burgeoning field with potential future applications in aquaculture. Further advancements in gut microbiota engineering techniques, coupled with artificial intelligence (AI) and synthetic biology, can drive substantial progress in sustainable aquaculture (Kumar et al., 2022). The establishment of comprehensive protocols and rigorous training programs encompassing advanced strategies and addressing safety considerations is paramount for the successful translation of microbiota engineering to real world applications. Given its capacity to bolster aquaculture sustainability through enhanced productivity, disease resilience, and ecological equilibrium, microbiota engineering emerges as a resilient and forward-looking strategy for advancing sustainable aquaculture practices.
